# Insights into Fast-Charge-Induced Cracking and Bulk Structural Deterioration of Ni-Rich Layered Cathodes for Lithium-Ion Batteries

**DOI:** 10.1021/acsnano.5c07347

**Published:** 2025-09-11

**Authors:** Jingyi Qu, Zhong Xie, Isobel C. Bicket, Hui Yuan, Lucia Zuin, Milenka Andelic, Wei Qu, Gianluigi A. Botton, Hanshuo Liu

**Affiliations:** † Department of Materials Science and Engineering, 3710 McMaster University, Hamilton, Ontario L8S 4L7, Canada; ‡ Clean Energy Innovation Research Centre, 6356 National Research Council Canada, Vancouver, British Columbia V6T 1W5, Canada; § Canadian Centre for Electron Microscopy, McMaster University, Hamilton, Ontario L8S 4M1, Canada; ∥ 117197 Canadian Light Source, Saskatoon, Saskatchewan S7N 2 V3, Canada

**Keywords:** Li-ion batteries, Ni-rich layered cathode, fast charge, cracking, cathode electrolyte interphase, Li loss, cation mixing

## Abstract

Ni-rich NMCs (lithium nickel manganese cobalt oxides) have been extensively utilized as a type of cathode material for Li-ion batteries due to their high energy density and cost efficiency. Meeting the increasing consumer demand for fast charging has become an urgent priority within the industry. Studies on structural degradation caused by fast charging, however, remain limited, especially concerning the understanding of the inter-related failure phenomena and mechanisms. In this study, our results reveal that prolonged fast-charge cycling of an NMC811 cathode leads to significant crack growth at both nano- and microscales. The rapid propagation of cracks from the particle interior to the surface significantly accelerates electrolyte infiltration, leading to the formation of the cathode electrolyte interphase inside NMC811 particles. A fatigue-cracking mechanism based on Li concentration gradients is proposed to be the root cause of crack formation. Additionally, electron energy loss spectroscopy and X-ray diffraction analysis provide direct evidence of irreversible Li loss and layered structure distortion in the bulk material of fast-charged NMC811, which contributes to the significant capacity loss of the NMC811 cathode after fast-charge cycling. The intragranular transition metal/Li cation mixing is also observed within the particle interior of fast-charged NMC811, which further deteriorates the material. This study offers valuable insights into the structural challenges encountered by Ni-rich NMC cathodes under fast-charge conditions, providing a foundational framework for designing strategies to enhance their structural integrity and electrochemical performance in demanding applications.

## Graphical abstract

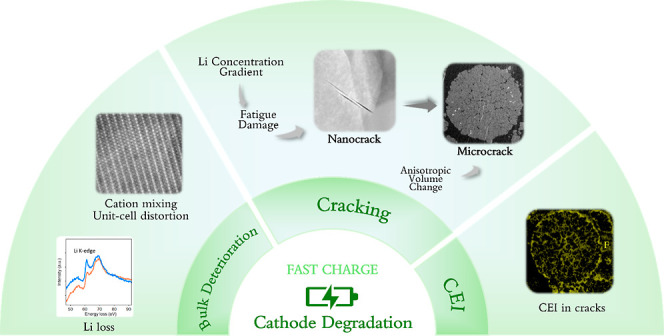

## Introduction

Over the past decades, there have been different generations of cathode materials utilized in lithium-ion batteries (LIBs) as the electric vehicle (EV) industry grows. Among many cathode candidates, lithium ternary transition-metal oxides, specifically nickel-rich lithium nickel manganese cobalt oxides (LiNi_*x*_Co_*y*_Mn_1–*x*–*y*_O_2_, *x* > 0.5), commonly referred to as Ni-rich NMC, are currently of the utmost interest for commercial LIB applications due to their high specific capacity.^1^ Despite the widespread use of Ni-rich NMC, the degradation mechanisms under some specific cycling conditions are not yet fully understood; thus, research is ongoing to further enhance their performance. To meet consumer demands for EVs, achieving fast-charge is a critical objective. However, this goal faces significant challenges while maintaining the capacity and cycling durability of LIBs with higher cycling rates.^2–6^ Extensive studies have examined the degradation mechanisms of LIB anodes during fast-charge.^7,8^ Issues such as lithium plating,^9–11^ heterogeneity,^12,13^ and solid electrolyte interphase (SEI) formation^14,15^ have been proposed as major causes of anode material degradation. However, despite their important role, research on the detailed structure of cathodes in general and Ni-rich NMC cathodes in particular under fast-charge conditions remains limited.

There have been a few studies investigating the structural evolution of NMC cathode materials under high cycling rates.^16–19^ Wang et al.^17^ investigated the rate-dependent phase evolution of NMC532 under fast cycling and proposed that the intermediate phase during the phase transition process is attributed to the uneven Li distribution induced by high current density. Similarly, the Li compositional heterogeneity induced by high-current cycling is also revealed to be the cause of unexpected phase transformation behavior, as claimed by Hyun et al.^18^ Furthermore, Tang et al.^19^ looked into the structural degradation of Ni-rich NMC under extreme fast cycling conditions and found that the NMC811 experienced severer *c*-axis change and more particle pulverization with a higher charge rate after prolonged cycling. Although the phase transformation has been reported in previous studies, investigation of the localized structural and compositional changes, especially the Li, in the particle bulk of Ni-rich NMC cathodes after fast-charge remains limited. It is also noteworthy that some of the studies were carried out on fast cycling (charge and discharge) conditions, where the structural evolution observed is from a combination effect of both fast-charge and fast-discharge without isolating the effects of fast-charge.

Cracking has been reported as an influencing factor that limits the rate capability of polycrystalline Ni-rich NMC cathodes.^20–24^ However, microcrack formation in NMC cathodes upon fast-charge remains controversial. Tanim et al.^25^ demonstrated that microcrack formation in the Ni-rich NMC cathode is more severe at a 1C charge rate compared to faster charge rates (4C and 9C), whereas some other studies^19,26^ reported that Ni-rich NMC cathodes experienced more microcracks when cycled under fast cycling rates comparing to slower ones. For the above-mentioned cases, the formation of microcracks is either attributed to the anisotropic unit-cell volume change or not thoroughly discussed. Therefore, the fundamental mechanisms of crack initiation and growth under high charge rates remain insufficiently understood.

In this work, we studied the charge-rate-dependent impact on the structure and chemistry of the Ni-rich LiNi_0.8_Mn_0.1_Co_0.1_O_2_ (NMC811) cathode in LIBs. The cathodes were cycled under different charge rates and a constant slow discharge rate in order to decouple the influence from fast-charge. Through electrochemical performance characterization of NMC811 cathodes subjected to varying charge rates, a significant irreversible capacity loss has been observed after prolonged cycling under faster charge conditions. To elucidate the underlying degradation mechanisms, an in-depth study has been conducted from the secondary particle level to the interior structure of primary particles, focusing on crack formation and propagation, cathode electrolyte interface (CEI) development, and bulk structural deterioration. Multiscale electron microscopy and spectroscopy characterization techniques including focused-ion beam (FIB), scanning electron microscopy (SEM), scanning transmission electron microscopy (STEM), electron energy loss spectroscopy (EELS), X-ray diffraction (XRD), and X-ray absorption spectroscopy (XAS) were employed. This multifaceted approach provides critical insights into the interplay between the aforementioned factors and their cumulative impact on the long-term performance of NMC811 cathodes under demanding fast-charge applications. The results reveal the irreversible lattice distortion in the bulk scale and atomic level associated with Li loss under fast-charge conditions.

## Results

### Cycling Performance of NMC811 with Different Charge Rates

The NMC811 half-cells were cycled under 0.2C, 0.5C, 1C, and 2C charge rates and 0.2C discharge rate for 200 cycles within the range of 3.0–4.3 V. This study is focused on the investigation of cathode evolution, so a half-cell configuration has been used to eliminate the influence from the graphite anode. It can be seen that the capacity retention decreases rapidly with the increase of charge rates from 0.2C to 2C during 200 cycles (cycling profiles in Figure S1). Then, the cells that underwent 200 cycles were set for the same slow rate (0.2C) charge/discharge cycling to evaluate their irreversible capacity loss. Figure 1a shows the charge/discharge curves of cycled NMC811 cathodes at 0.2C cycling rate. The results demonstrate that the 0.2C-charged, 0.5C-charged, and 1C-charged NMC811 cathodes experience a small amount of irreversible capacity loss, with discharge capacities of 147, 139, and 135 mAh/g, indicating a capacity loss of about 4%, 9%, and 12%, respectively, compared to the initial cycle discharge capacity of the 0.2C charge/discharge NMC811 cell. Significant irreversible capacity loss is observed from the 2C-charged cathode, where only 107 mAh/g of discharge capacity has been restored, indicating that the 2C-charged NMC811 cathode experienced dramatic irreversible degradation.

**Figure 1:**
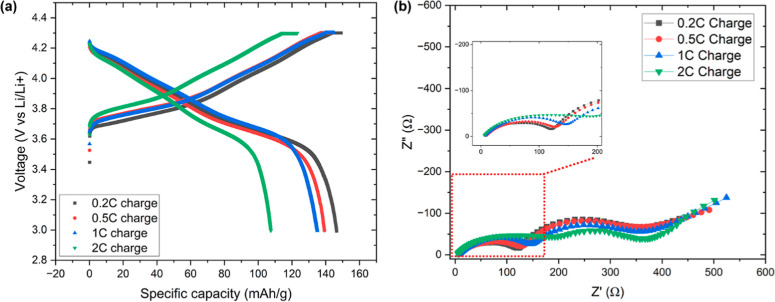
(a) Cycling profiles of cycled NMC811 cathodes at 0.2C charge/discharge rate. The cathodes have experienced 200 cycles at different charge rates. (b) Nyquist plot of cycled NMC811 cathodes after 200 cycles at different charge rates.

Figure 1b shows electrochemical impedance spectroscopy (EIS) results for NMC811 half-cells after 200 cycles at various charge rates. It can be seen that the high-frequency semicircle grows larger with the increase of the charge rate from 0.2C to 2C. Since the high-frequency semicircle is correlated with the electrode’s surface film resistance (*R*_f_), the larger *R*_f_ of higher-rate charged NMC811 cathodes, especially the 2C-charged cathode, could be ascribed to the formation of a thicker CEI layer compared to the 0.2C-charged cathode. Additionally, the 2C-charged NMC811 cathode also exhibits a smaller semicircle at medium frequencies compared to the other three slower-rate charged cathodes. The semicircle at medium frequencies corresponds to the charge transfer process of the cell. This result therefore indicates a smaller charge transfer resistance (*R*_ct_) for the 2C-charged cathode. As *R*_ct_ reflects the kinetic hindrance of redox reactions at the electrode and electrolyte interface, the difference observed in the *R*_ct_ may be closely associated with the different electrode/electrolyte interface configuration of cycled NMC811 cathodes. More details regarding the microstructure evolution of cycled NMC811 cathodes will be discussed below to understand the correlation of electrochemical performance with the microstructure.

### Microcrack and CEI Formation

As shown in Figure 2a–d, there are noticeable differences in microcrack generation of cycled NMC811 cathodes under different charge rates. The 0.2C-charged NMC811 cathode (Figure 2a and S2b) exhibits minimal microstructural evolution compared with the pristine cathode (Figure S2a), with no visible microcracks observed. For the 0.5C- and 1C-charged NMC811 cathodes, however, some premature microcracks were observed near the center of secondary particles with the 1C-charged NMC811 cathode (Figure 2c and S2d) exhibiting a slightly higher level of cracking than the 0.5C-charged cathode (Figure 2b and S2c). More significant microcrack formation is observed from the 2C-charged NMC811 cathode, as shown in Figure 2d and S2e, where the microcracks were fully developed.

**Figure 2:**
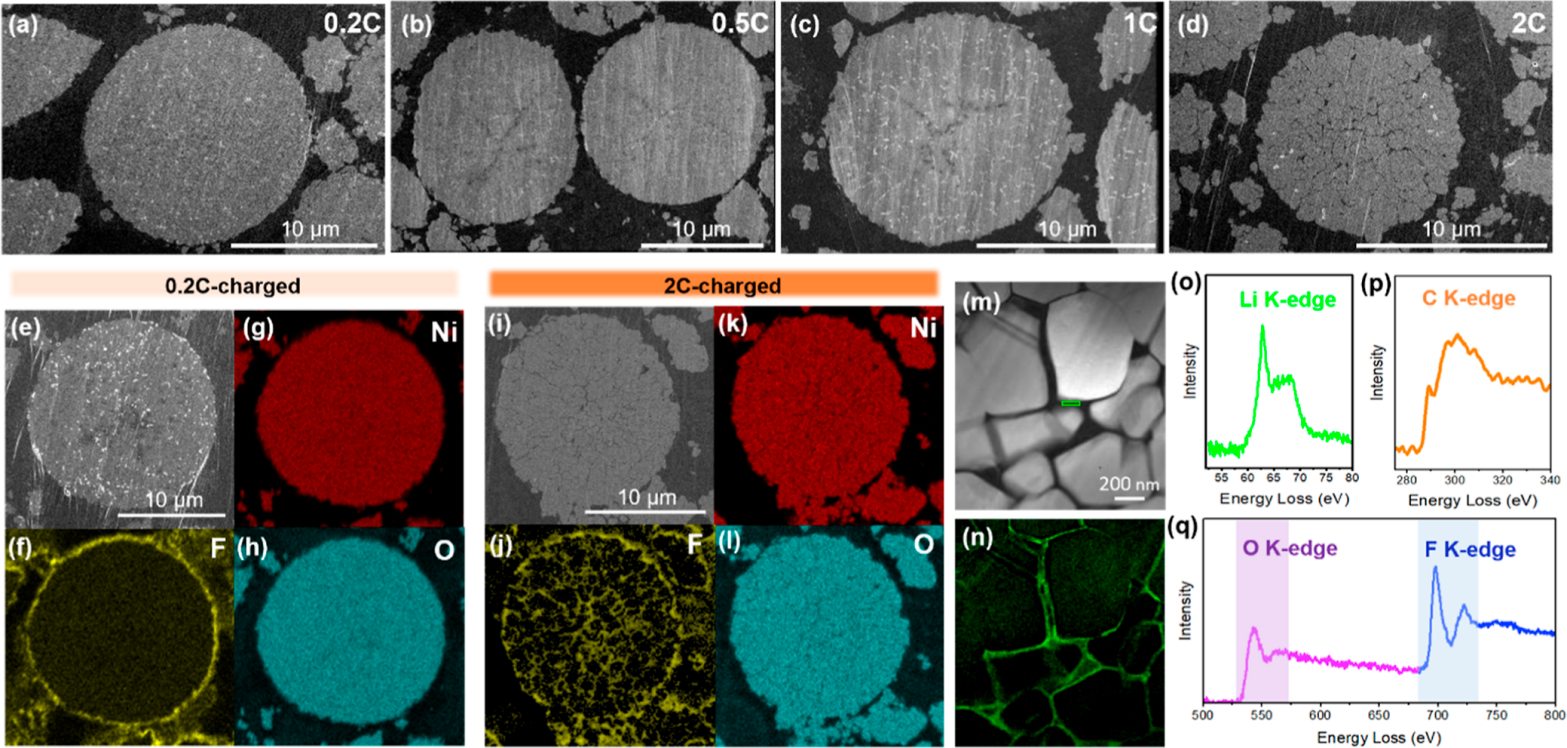
Cross-sectional SEM images of (a) 0.2C-charged, (b) 0.5C-charged, (c) 1C-charged, and (d) 2C-charged NMC811 cathodes. (e) Cross-sectional SEM image of the 0.2C-charged NMC811 cathode with corresponding (f) F, (g) Ni, and (h) O EDX maps. (i) Cross-sectional SEM image of the 2C-charged NMC811 cathode with corresponding (j) F, (k) Ni, and (l) O EDX maps. (m) A higher-magnification STEM image of the 2C-charged NMC811 cathode. (n) Li K-edge MLLS map with the Li K-edge reference spectrum in (o). EELS spectra collected from the rectangle-marked CEI region in (m), including (o) Li K-edge, (p) C K-edge, and (q) O K-edge and F K-edge.

The SEM–EDX analysis further reveals the formation of CEI surrounding and within the cycled NMC811 secondary particles. Figure 2f–h shows the F, Ni, and O EDX maps for the 0.2C-charged NMC811 cathode, and Figure 2j–l shows the 2C-charged cathode. As shown in Figure 2f, the surface of the 0.2C-charged NMC811 secondary particle was covered by a fluorine (F)-containing layer, which is not observed in the pristine cathode (Figure S3). This is due to the formation of CEI around the particle surface, which contains F-based species from electrolyte decomposition, e.g., LiF. Note that the cycled cathodes were washed with dimethyl carbonate (DMC) prior to the sample preparation, in order to remove the residual electrolyte salt. In contrast to the 0.2C-charged NMC811 secondary particles, where F is only detected from the surrounding surface, the 2C-charged NMC811 also contains F within the particle interior (Figure 2j), indicating the penetration of the electrolyte into NMC811 secondary particles and the subsequent growth of CEI along the microcracks. Figure S4 shows the Li K-edge of the 2C-charged NMC811 cathode, collected by synchrotron XAS in total electron yield (TEY) mode. The Li K-edge spectrum can be identified as LiF according to the literature.^27^ The detection depth for the TEY mode is approximately 10 nm, indicating a minimum thickness of 10 nm for the CEI.

Figure 2m is a higher-magnification cross-sectional STEM image showing multiple primary particles of a 2C-charged NMC811 secondary particle. From the same area, Figure 2n presents a Li K-edge multiple linear least-squares (MLLS) map derived from fitting the Li K-edge reference spectrum shown in Figure 2o. This analysis corresponds to the region depicted in Figure 2m and reveals the spatial distribution of the CEI layer surrounding the primary particles inside the NMC811 particle. A CEI region between two primary particles is marked with a green rectangle and selected for further spectral analysis. Figure 2o–q show the EELS spectra extracted from the green rectangle marked CEI region, including Li K-edge, C K-edge, O K-edge, and F K-edge, which arise from electrolyte decomposition products, such as, LiF, Li_2_CO_3_, etc.^28–30^

The results of the fast-charged NMC811 suggest that the microcracks are connected from the particle interior to the surface. This observation is consistent with the decreased *R*_ct_ observed from EIS results for the 2C-charged sample. The well-connected microcracks could lead to electrolyte infiltration and therefore an increase in the electrolyte and electrode interface, which would accelerate the charge transfer process to some extent.

Overall, the cross-sectional SEM images and EDX maps demonstrate that more microcracks are generated as the charge rate increases from 0.2C to 2C, with a significant increase observed at the 2C charge rate. The observed trend in microcrack formation across varying charge rates corresponds closely with the irreversible capacity loss after extended cycling (Figure 1a), where a more prominent capacity fade is observed in the 2C-charged cathode. The results therefore suggest that the generation of microcracks and the formation of CEI within NMC811 secondary particles would be main contributors to the irreversible capacity loss of NMC811 during fast-charge.

### Intragranular Nanocrack Formation

In addition to the microcracks discussed above, intragranular nanocracks are also observed within the primary particles of the 2C-charged cathode. Figure 3a–e and S5 show high-angle annular dark-field (HAADF)-STEM images of the 2C-charged NMC811 cathode. The contrast of a HAADF-STEM image is proportional to the atomic number of the elements in the materials, where elements with higher atomic numbers appear with brighter intensity (e.g., transition metals (TMs)), and light elements, such as Li, are not visible from the image. As can be seen from Figure 3a, intragranular nanocracks with sizes of a few nanometers in width and ∼50–100 nm in length are formed inside the primary particles of the 2C-charged NMC811 cathode. Some premature nanocracks are also observed near the surface, as shown in Figure 3b. The formation of these intragranular nanocracks in 2C-charged NMC811 is closely related to the uneven Li distribution inside particles during fast-charge cycling. Under a higher charge rate, with more Li ions being extracted from the particle surface, a larger Li concentration gradient will be formed during delithiation. The difference in Li content will result in different levels of unit cell expansion along the *c*-axis inside the particle, with a larger *c*-axis expansion near the surface. This will lead to the particle surface experiencing significant tensile stress which rapidly reduces toward the particle interior, with the core undergoing compression, as demonstrated in the sketch of Figure 3f. This process repeats during each fast-charge cycle until the material eventually fractures and crack growth occurs. This experimental finding is consistent with theoretical simulations found in the literature.^31^ The influence of the C-rate on fatigue cracking has been simulated through multiphysics phase field modeling, demonstrating that high C-rates accelerate the growth of fatigue cracks, where the number of cycles required for unstable crack growth is much lower for high C-rates due to the higher stress magnitude arising from a larger Li concentration gradient.

**Figure 3:**
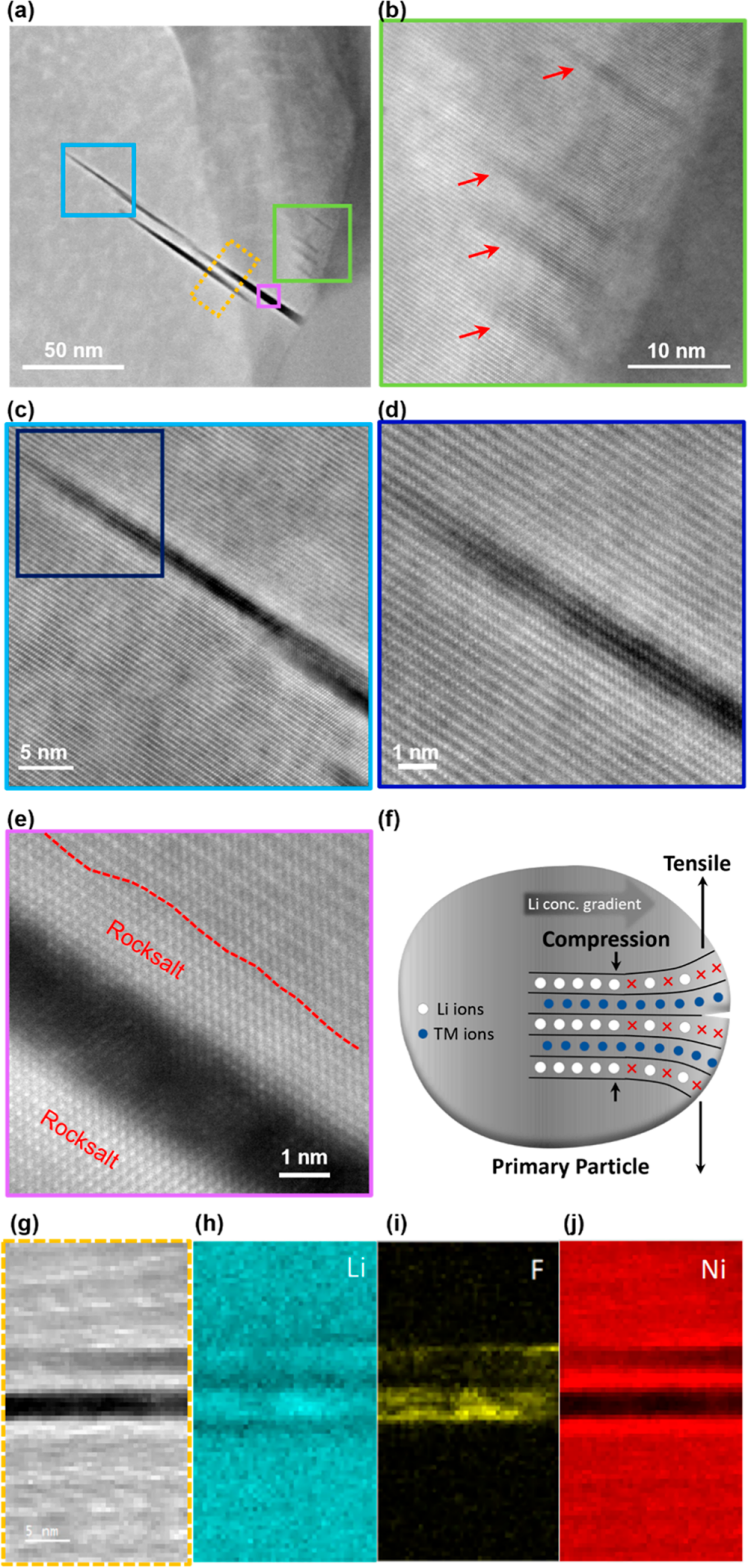
Intragranular nanocrack characterization. (a) HAADF-STEM image of the 2C-charged NMC811 cathode with several intragranular nanocracks. (b–e) Enlarged HAADF-STEM images of regions marked in (a). (f) Schematic illustration of the generation of an intragranular nanocrack. (g) EELS spectrum image of an intragranular nanocrack and the corresponding (h) Li, (I) F, and (j) Ni EELS maps.

Additionally, the atomic-resolution HAADF-STEM images (Figure 3c–e) also show that the growth direction of intragranular nanocracks is within the TM and Li lattice planes (ab-plane) and perpendicular to the *c*-axis. This observation aligns with the explanation of nanocrack formation in which the fast-charged NMCs experience high tensile stress near the surface along the *c*-axis. First-principles calculations also show that the NMCs exhibit anisotropy in elasticity during delithiation, with a significant reduction in elasticity along the *c*-axis.^32^ This reduction makes the material more prone to bond breaking perpendicular to the *c*-axis when tensile stress is applied, leading to crack formation along the ab-plane.

Interestingly, it is worth noting that a Li-rich region is observed within the nanocrack, along with the presence of F (Figure 3h–j), indicating that the formation of CEI occurs within the nanocrack. The observation of CEI inside the primary particles suggests that the nanocracks are not enclosed but are exposed to the particle surface or connected to the microcracks, through which the electrolyte has infiltrated the primary particles. Furthermore, a disordered rock salt-like phase^33^ is observed near some of the nanocrack regions, as shown in Figure 3e. This is most likely due to the phase transformation arising from the infiltration of the electrolyte into the fresh nanocracks during cycling. The newly exposed nanocrack-induced surface underwent a phase transformation associated with TM migration, which is a well-recognized degradation mechanism of Ni-rich NMC cathodes.^28^ The formation of the rock salt-like phase is consistent with the Ni segregation observed at the nanocrack region from the EELS map (Figure 3j). The formation of this disordered phase near nanocracks could impede Li transport and induce local strain, which further promotes crack expansion and cathode material degradation.

It is also noticed that not all the surface around the nanocracks transformed into the rock salt-like phase, as shown in Figure 3d. This may be due to the duration of electrolyte exposure, since the nanocrack surface regions that maintained the layered structure are closer to the crack tip, where the surfaces are likely relatively fresh and the phase transformation has not yet occurred. For reference, Figure S6 shows the intact morphology of pristine NMC primary particles without any intragranular nanocrack. This confirms that the formation of nanocracks is a result of electrochemical cycling that should not be simply attributed to the synthesis or sample preparation.

### Bulk Structure Degradation

To further investigate the structural evolution of fast-charged NMC811, we conducted XRD characterization. The XRD patterns and refinement results (detailed crystallographic parameters in Tables S1–S5) of pristine and cycled NMC811 cathodes are presented in Figure 4. After 200 cycles, the NMC811 cathodes maintain a layered structure for the bulk material. The unit cell parameters exhibit an increase in the *c*-axis and a decrease in the *a*-axis with the increase of charge rates from 0.2C to 2C, as depicted in Figure 4b–d. The expansion of the *c*-axis in NMC layered cathodes is attributed to the repulsion between oxygen layers during delithiation, when fewer Li ions are contained in the Li layers.^19,34–36^ Therefore, the expansion of the *c*-axis suggests that the NMC811 cathodes experience more Li loss as the charge rate increases. The shrinkage of the *a*-axis could be due to TM oxidation with decreased ionic radii,^37^ suggesting potential charge compensation due to Li loss. The volume change follows the trend of the *a*-axis change. Since the unit cell volume is proportional to the square of “a”, the influence of the *a*-axis is reflected more strongly in the volume change. The ratio of c/a has been used to examine the layered structure characteristics^38^ and can also be seen as an indicator reflecting the hexagonal (unit cell) distortion. As shown in Figure 4e, the c/3a ratio of cycled NMC811 increases with the increase of the charge rate, indicating that NMC811 after 2C-charged cycling suffers a higher level of unit cell distortion, which is correlated with the intense microcrack development of the 2C-charged NMC811 cathode.

**Figure 4:**
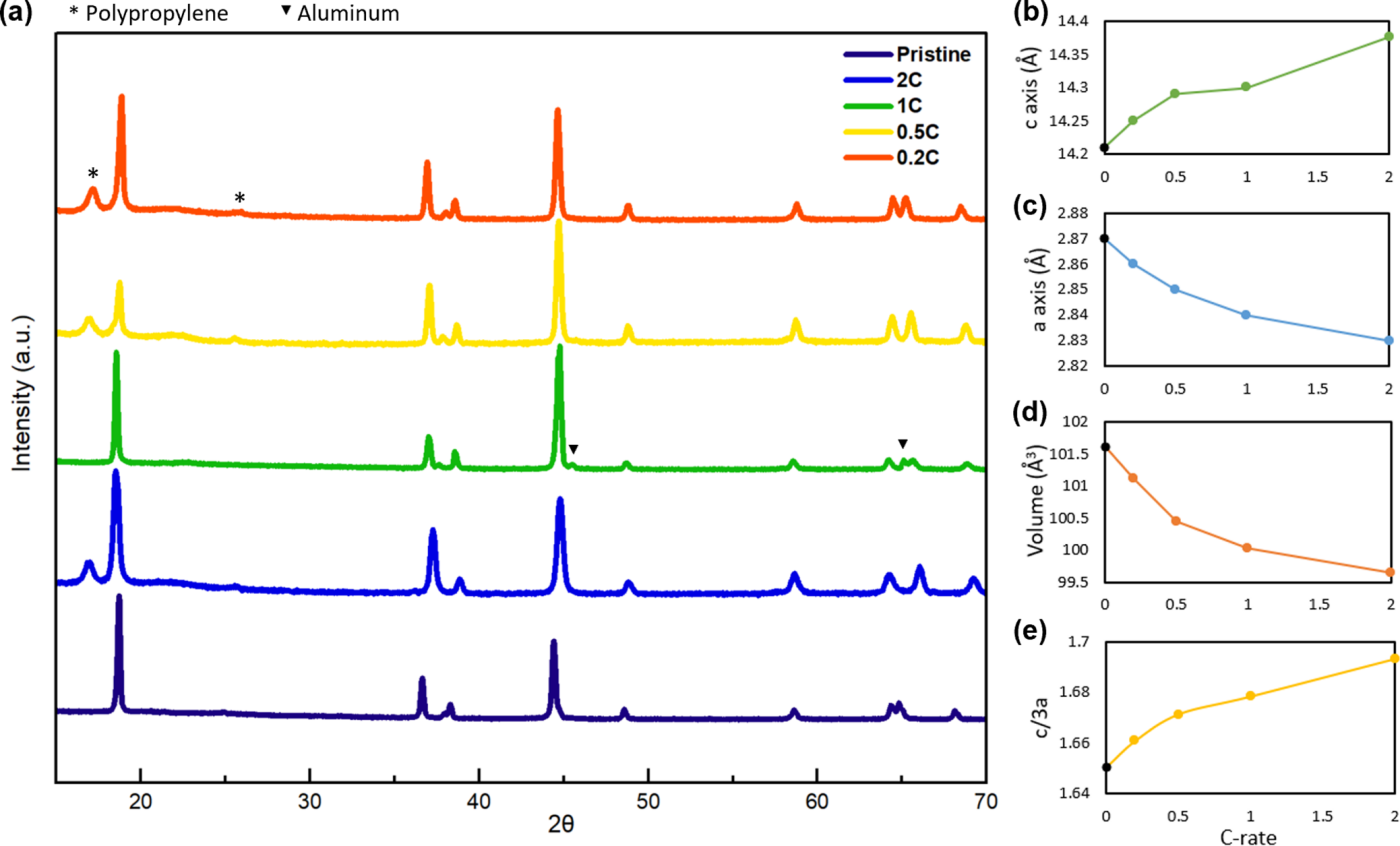
(a) XRD patterns of pristine, 0.2C, 0.5C, 1C, and 2C-charged NMC811 cathodes. Refinement results for the (b) *c*-axis, (c) *a*-axis, (d) volume, and (e) c/3a ratio. (Note: the additional small peaks, distinct from the main layered phase, arise from the separator or the aluminum current collector, have been labeled accordingly.)

As shown in the HAADF-STEM images (Figure 5a,b), pristine NMC811 exhibits a well-defined layered structure. In comparison, some disordered domains with TM/Li cation mixing are observed in the bulk of the 2C-charged NMC811, as shown in Figure 5d,e and S7. In the Z-contrast HAADF-STEM images, brighter TM atoms were observed in the Li layers, indicating the presence of TM atoms in Li planes. This is further confirmed by the intensity line profile shown in Figure 5f. The weak peaks (indicated by the black arrows) between the TM plane peaks represent the Li/TM mixed planes, indicating the migration of TM atoms into Li sites during fast-charge. This observation indicates the presence of TM/Li cation mixing, where some TM atoms have migrated to Li sites during fast-charge cycling. This fast-charge-induced structural lattice distortion could impede Li transport inside primary particles and introduce local strain, which could promote further material degradation.

**Figure 5:**
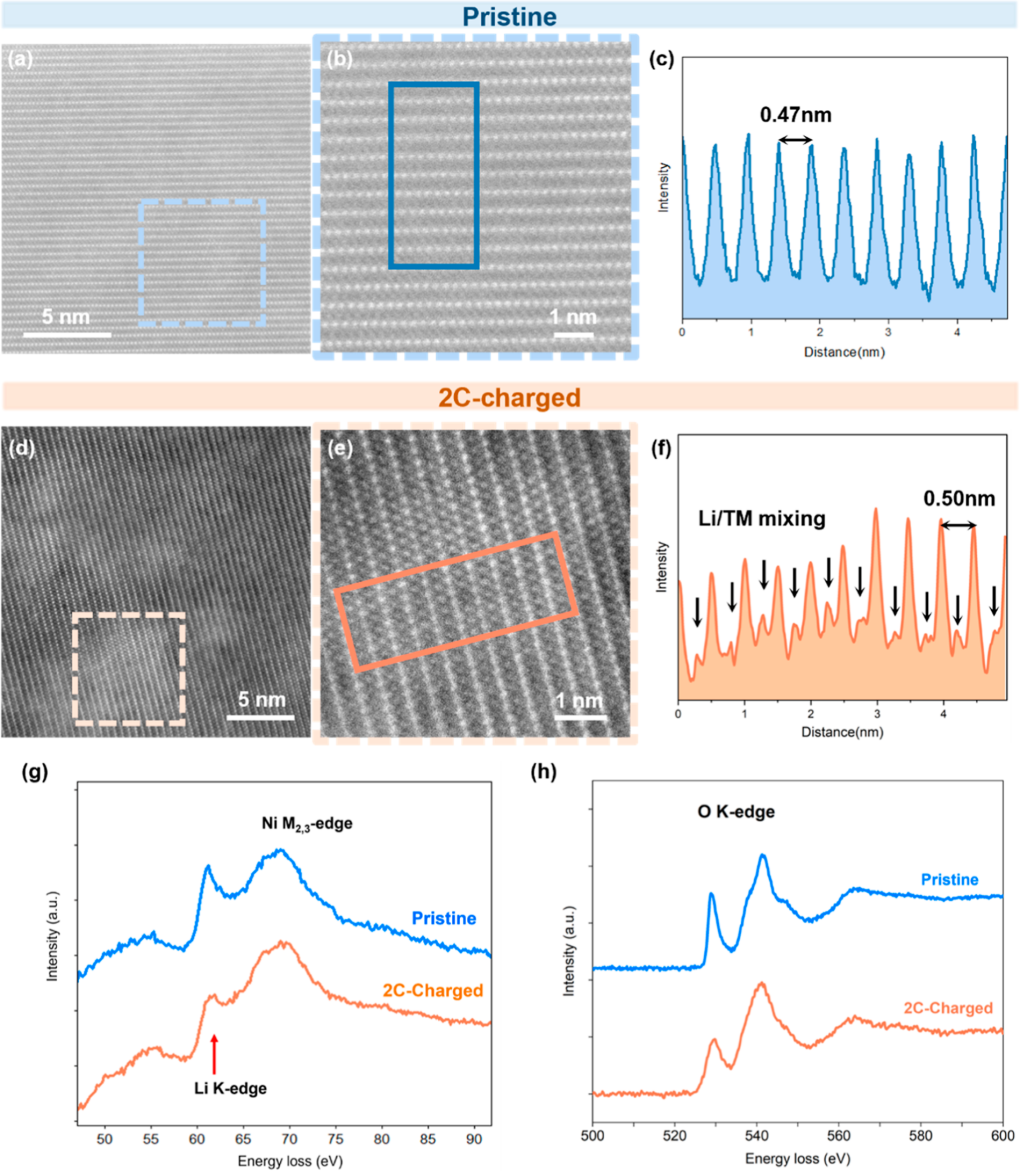
(a,b) High-resolution STEM images showing the bulk structure of the pristine NMC811 cathode. (c) Intensity line profile of the rectangular region marked in (b). (d,e) High-resolution STEM images showing the bulk structure of the 2C-charged NMC811 cathode. (f) Intensity line profile of the rectangular region marked in (e). EELS spectra of (g) Li K-edge and (h) O K-edge of pristine and 2C-charged NMC811 cathodes.

The Li K-edge EELS spectrum collected from the bulk of 2C-charged NMC811 also exhibits a lower peak intensity compared to the pristine NMC (Figure 5g), suggesting irreversible Li loss in the bulk material of the 2C-charged cathode. As discussed above, with less Li in the Li plane, the O–O Coulombic repulsion between adjacent planes will lead to unit cell expansion along the *c*-axis, which is consistent with the *c*-axis elongation observed from XRD refinement results. Meanwhile, this irreversible loss of Li inventory would be a main contributor to the capacity loss of 2C-charged NMC811 after 200 cycles, as discussed in Figure 1b. Furthermore, a decrease in the O K-edge prepeak intensity (indicated by the arrow) of 2C-charged NMC811 compared to the pristine NMC811 is observed in the bulk, as shown in Figure 5h. The O K-edge prepeak originates from electron transitions from O 1s states to unoccupied O 2p and TM 3d hybridized states. The decreased intensity indicates a reduced O contribution to the TM_3d_-O_2p_ hybridization orbital, with potential oxygen release.^39^ The phase transition occurring at highly delithiated states in Ni-rich cathodes has been reported to trigger oxygen release.^40^ During prolonged fast-charge cycles, the nonuniform distribution of Li is believed to result in locally delithiated regions at relatively high voltages, which are suggested to be the primary origin of oxygen loss. This finding is consistent with the literature, which suggests the concurrent removal of Li and O during fast charging.^41^

## Discussion

Since the formation and development of cracks are closely related to the rate performance, the results reveal that cracking is of primary concern in fast-charged Ni-rich NMC cathodes. Particle cracking is commonly proposed to originate from the large anisotropic unit cell expansion and contraction for polycrystalline particles.^42,43^ This phenomenon is more prominent when higher capacities are delivered, with a higher quantity of Li ions being extracted from and inserted into the cathode materials; for instance, cathodes cycled under high-voltage conditions. But this does not fully explain the case for fast charging. Since lower capacities are delivered under higher charge rates, fewer Li ions are being extracted from and inserted into the cathode materials. Therefore, the crack formation and growth of NMC cathodes under fast-charge conditions cannot be simply explained by the anisotropic unit cell volume change. As discussed above, this effect is closely related to the large Li concentration gradient induced by the high charge rate and the resultant stress near the surface and core.

Meanwhile, it is worth noting that the polycrystalline NMC particles still suffer from anisotropic unit cell expansion under fast-charge conditions, which would also contribute to microcrack growth. Single crystalline materials have been proposed to solve this issue.^44,45^ While the single crystalline materials have the apparent advantage of mitigating anisotropic unit cell expansion and the resultant intergranular cracking, the intragranular nanocracks formed inside primary particles generated from the Li concentration gradient under fast-charge may still be present in the single crystalline particles. The results, therefore, suggest that one key point in preventing crack formation under fast-charge conditions would be to enhance the internal Li diffusion kinetics of the active material and provide good conductive pathways in the electrode in order to reduce the Li concentration gradient generated during high charge rates.

The growth of cracks also facilitates electrolyte infiltration into the active material, leading to CEI formation inside NMC particles and a layered-to-rock salt phase transformation at the crack-induced surface, which contributes to cathode performance decay. Furthermore, fast charging also leads to Li loss from the bulk material and the formation of disordered TM/Li domains. The migration of TM atoms into Li sites will block the Li pathway, hindering Li transport and resulting in Li loss. Additionally, the fast-charge-induced disordered TM/Li structure causes lattice distortion, leading to local strain during charge and discharge, which can accelerate further structure degradation and crack formation. The irreversible loss of the Li inventory would lead to capacity fade. The overall degradation process of fast-charge NMC811 is summarized in Figure 6.

**Figure 6:**
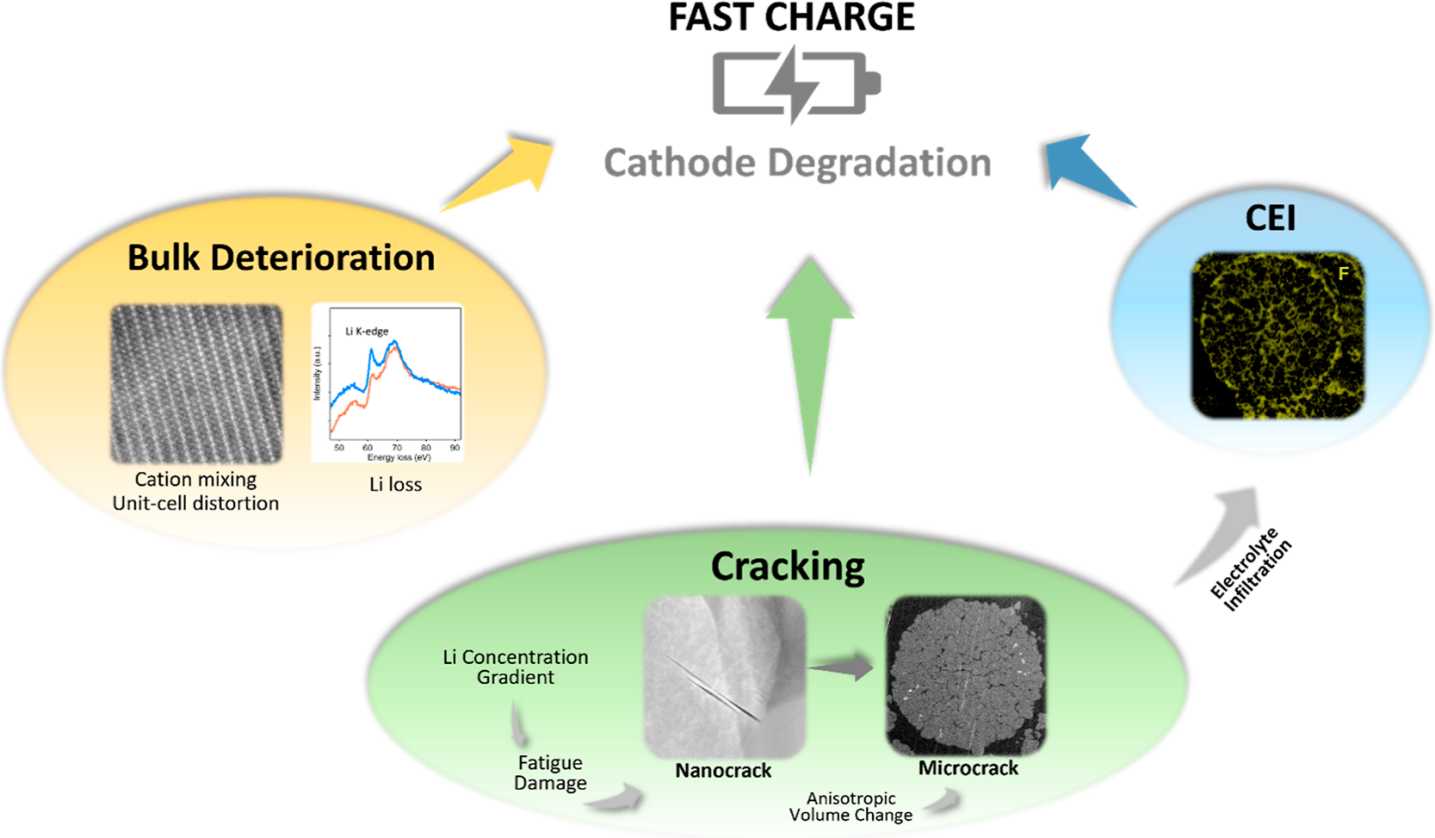
Schematic illustration of the degradation mechanism of the NMC811 cathode under fast-charge condition.

## Conclusions

In this work, an in-depth study was conducted on the fast-charge-induced material degradation of the Ni-rich NMC811 cathode. The fatigue cracking and bulk phase evolution are the primary factors that cause the deterioration of fast-charge cathodes. The results indicate that fast-charge-induced crack formation is not only attributed to the conventional anisotropic unit cell volume change mechanism; another dominant factor is the fatigue damage experienced by the NMC particles under repeated tensile and compression stresses at the particle surface and core, driven by the large Li concentration gradient formed during fast-charge. The crack formation and growth deteriorate the integrity of the active materials, facilitating electrolyte infiltration into NMC particles. This promotes CEI formation and undesired phase transformation at the newly exposed crack surfaces, leading to an irreversible capacity loss. Furthermore, our results reveal that the fast-charged NMC experiences Li loss from the bulk material and intragranular TM/Li disordering within the particle interior, which further contributes to the irreversible capacity loss of NMC811 cathodes after fast-charge cycling and could facilitate further lattice distortion and crack propagation. The results provide an in-depth understanding of the structural degradation mechanism in Ni-rich NMC cathodes under fast-charge conditions, suggesting the strong correlation between materials’ Li diffusion properties and crack formation. Our findings offer fundamental guidance for the design and synthesis of cathode materials, emphasizing the enhancement of structural integrity to better accommodate fast-charge applications.

## Methods

### Preparation of Electrodes and Coin Cells

The NMC811 electrodes were prepared with 92.54 wt % NMC811 powder (MTI Corporation), 3.73 wt % Super P carbon (Timcal), and 3.73 wt % polyvinylidene fluoride (HSV900, Kynar). We will hereafter refer to the NMC811 electrode as our “cathode” whether we are speaking about its role in cell discharge or charge. The coin cells were assembled with the NMC811 cathode, a Li metal anode, a Celgard 2500 separator, and an electrolyte consisting of 1 M LiPF6 in 1:1 v/v EC/DEC with 10 wt % FEC in an Ar atmosphere glovebox.

### Electrochemical Characterization of NMC Cathodes

The coin cell testing was carried out on a Solartron 1470E multichannel potentiostat. The coin cells were conditioned at a C/20 rate for 2 cycles. Then, the cells were put under galvanostatic cycling between 3.0 V-4.3 V using a constant current-constant voltage charge and constant current discharge protocol with various charge rates of 0.2C, 0.5C, 1C, 2C and the same discharge rate of 0.2C at room temperature. After 200 cycles, the cells were cycled with a 0.2C charge/discharge rate to evaluate their irreversible capacity loss. The NMC811 cathodes were then collected from the disassembled coin cells, washed with dimethyl carbonate (DMC) and dried in a glovebox for further postanalysis. The electrochemical impedance spectroscopy (EIS) measurements of the coin cells were carried out using a Solartron 1260 frequency response analyzer (FRA) under a 10 mV root-mean-square (RMS) perturbation with the frequency range of 200 kHz–1 mHz at the fully discharged state.

### Structural Characterization of NMC Cathodes

The cross sections of NMC811 cathodes were prepared using JEOL (IB-19520CCP) ion beam cross section polisher at 5 kV. The scanning electron microscopy (SEM) imaging and energy-dispersive X-ray spectroscopy (EDX) analysis were conducted using an FEI Magellan 400 microscope: a high-resolution SEM equipped with a field emission gun (FEG) and two Oxford XMaxN 80 detectors. The crystallographic structures of pristine and cycled NMC811 were characterized by X-ray Diffraction (XRD) using a Bruker D8 Advanced automatic diffractometer with Cu Kα radiation. The refinement was processed by GSASII by the Rietveld method.

A Thermo Fisher Scientific Helios G4 plasma-focused ion beam (FIB) was utilized to prepare the TEM samples of the 2C-charged and pristine NMC cathodes. The TEM samples were precleaned by a Gatan Solarus Plasma Cleaner prior to the TEM sessions. The atomic-resolution HAADF-STEM imaging was conducted using a Thermo Fisher Spectra Ultra microscope, equipped with aberration correctors for the probe- and image-forming lenses and operated at 200 keV. For chemical analysis, EELS data were acquired using a Gatan Continuum spectrometer equipped with a K3 camera at dispersions of 0.18 and 0.09 eV/channel. For all of the EELS spectra shown in this work, the background prior to the edge was subtracted with a standard power-law model. MLLS fitting was employed to decompose the collected EELS spectra into contributions from known reference spectra. In our case, the reference spectra used for fitting were directly extracted from the experimental EELS spectrum image (SI). A model consisting of a linear combination of the specified reference spectra was used to fit the spectra acquired from the experiment. The fitting coefficients are adjusted to minimize the sum of squared residuals between the linearly fitted model and the selected spectrum.

Soft-XAS was carried out for the Li K-edge on the VLS-PGM beamline at the Canadian Light Source synchrotron. With synchrotron-based soft-XAS, depth-sensitive characteristics can be detected by using the total electron yield (TEY) acquisition mode with a microchannel plate detector. With the TEY mode, only the electron signals escaping from the outermost surface of the materials (∼10 nm) are detected. The slit size for the incident beam was 100 × 100 μm. The acquisition energy range for Li K-edge was 40 – 75 eV.

## Supplementary Material



## Notes

Cycling performance data, SEM and STEM characterization images, XAS spectrum, and XRD refinement parameters (PDF)
