# Supplementary material for: Insights into Fast-Charge-Induced Cracking and Bulk Structural Deterioration of Ni-Rich Layered Cathodes for Lithium-Ion Batteries
Source: ACS Nano. 2025 Sep 11;19(37):33202–11. doi: 10.1021/acsnano.5c07347 (PMC12462233; doi:10.1021/acsnano.5c07347)
Supplement: Supplementary file 1 [file nn5c07347_si_001.pdf]

Supporting Information

Insights into Fast-Charge Induced Cracking and Bulk Structural Deterioration of Ni-Rich Layered Cathode for Lithium-Ion Batteries

Jingyi Qu[1], Zhong Xie[2], Isobel C. Bicket[1], Hui Yuan[3], Lucia Zuin[4], Milenka Andelic[1], Wei Qu[2], Gianluigi A. Botton[1,4]* and Hanshuo Liu[2]*

1 Department of Materials Science and Engineering, McMaster University, Hamilton, Ontario L8S 4L7, Canada

2 Clean Energy Innovation Research Centre, National Research Council Canada, Vancouver, British Columbia V6T 1W5, Canada

3 Canadian Centre for Electron Microscopy, McMaster University, Hamilton, Ontario L8S 4M1, Canada

4 Canadian Light Source, Saskatoon, Saskatchewan S7N 2V3, Canada

*Corresponding authors: gbotton@mcmaster.ca; hanshuo.liu@nrc-cnrc.gc.ca

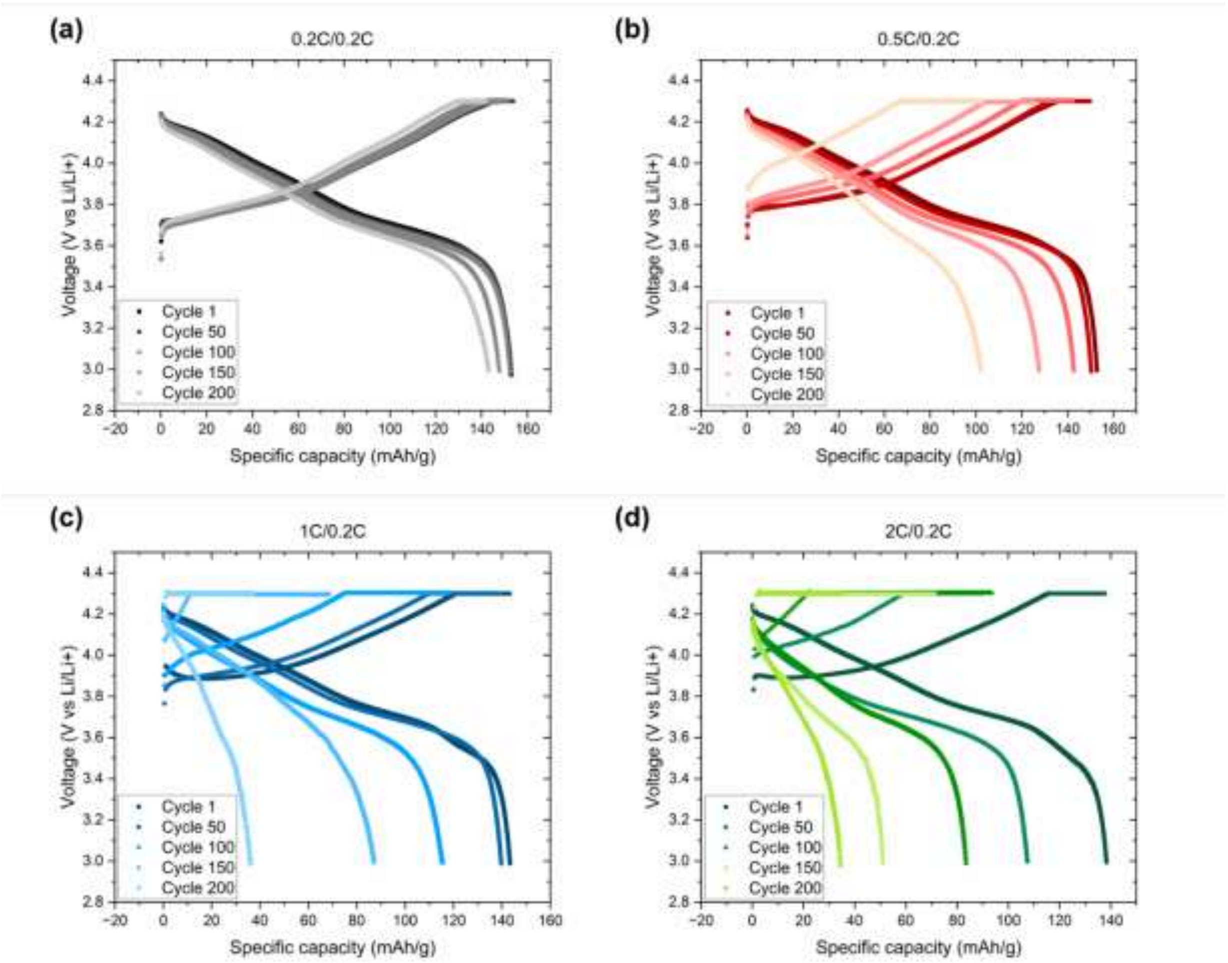

Figure S1 Galvanostatic charge-discharge profiles of cycled NMC811 cathodes at (a) 0.2C/0.2C; (b) 0.5C/0.2C; (c) 1C/0.2C; (d) 2C/0.2C charge/discharge rates for up to 200 cycles.

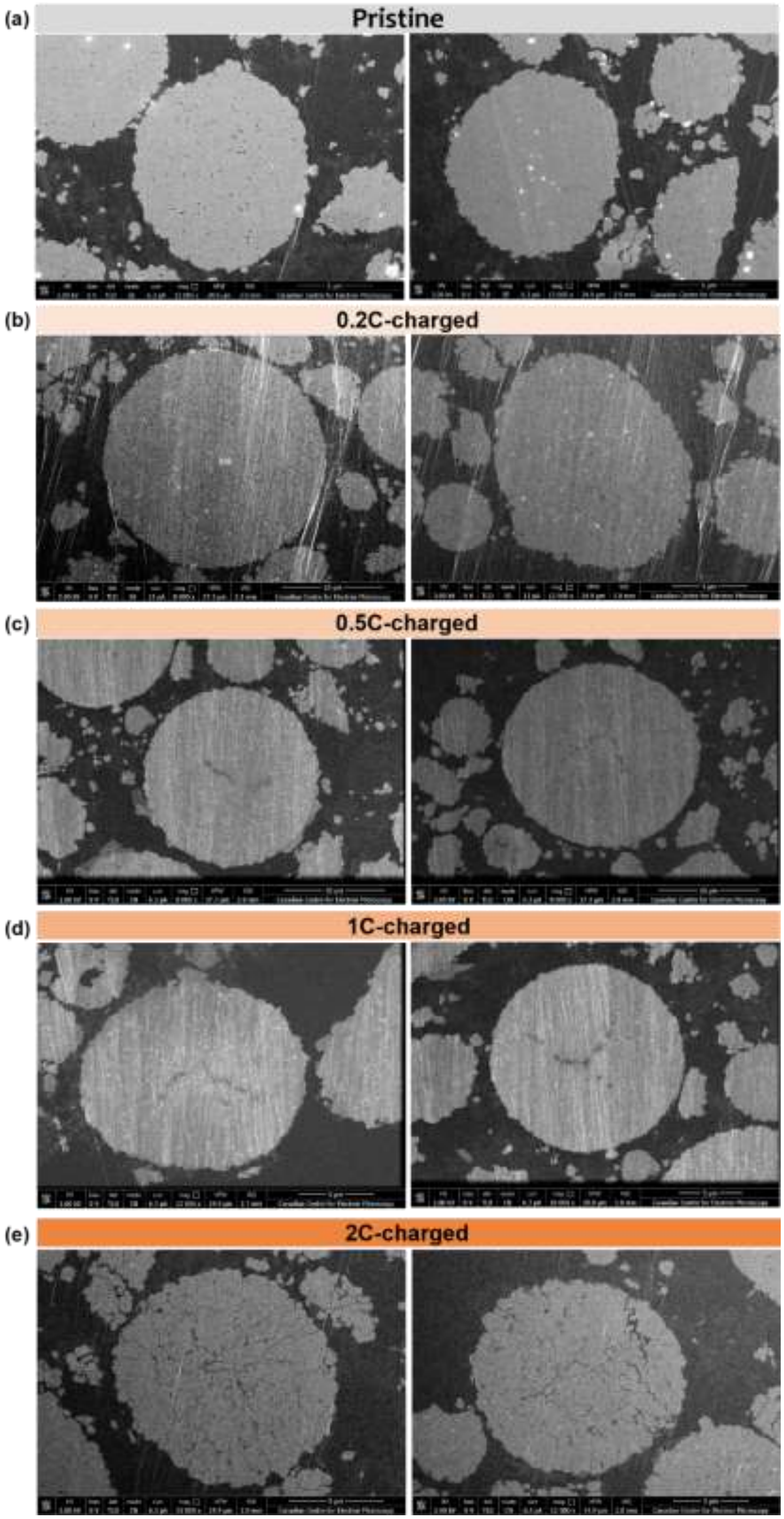

Figure S2 Cross-sectional SEM images of (a) pristine, (b) 0.2C-charged, (c) 0.5C-charged, (d) 1C-charged, (e) 2C-charged NMC811 cathodes.

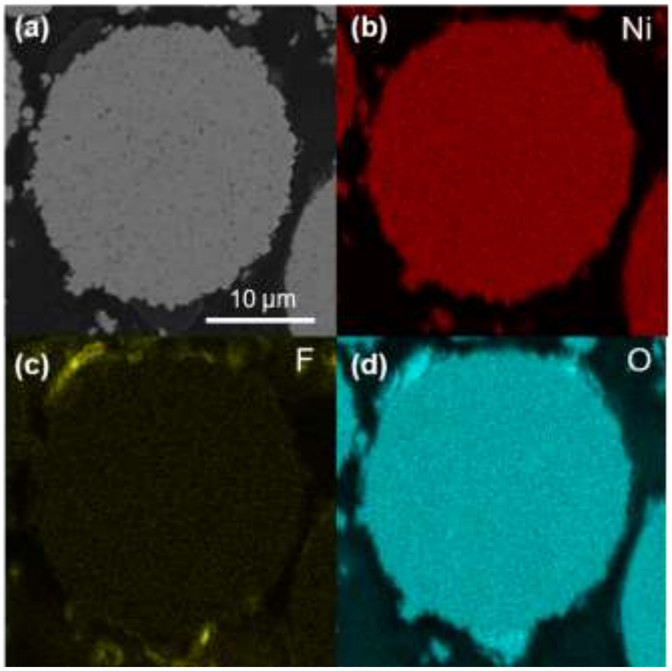

Figure S3 (a) Cross-sectional SEM image of pristine NMC811 cathode with corresponding (b) Ni (c) F (note that the small amount of F comes from PVDF binder) (d) O EDX maps.

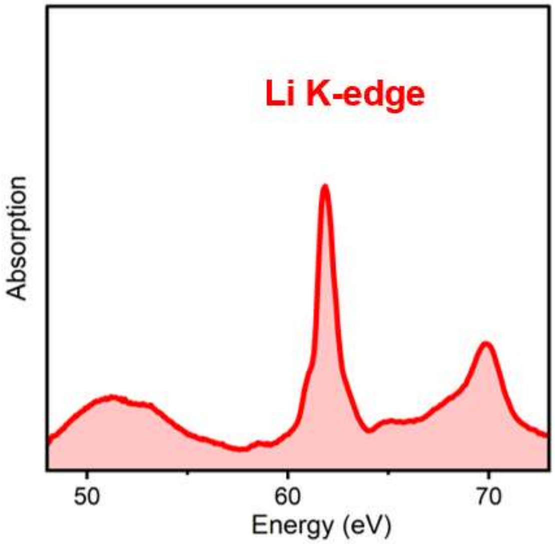

Figure S4 XAS Li K-edge spectrum of 2C-charged NMC811 cathode collected with TEY mode.

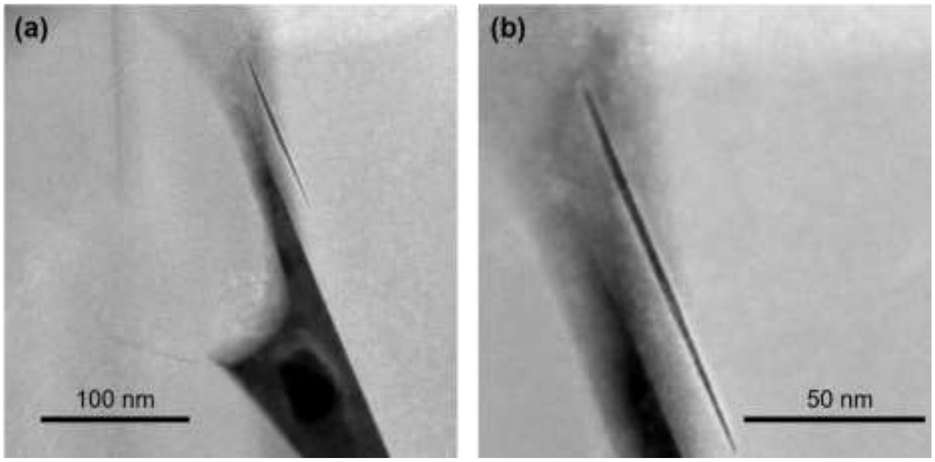

Figure S5 HAADF-STEM images of 2C-charged NMC811 cathode with intragranular nanocrack.

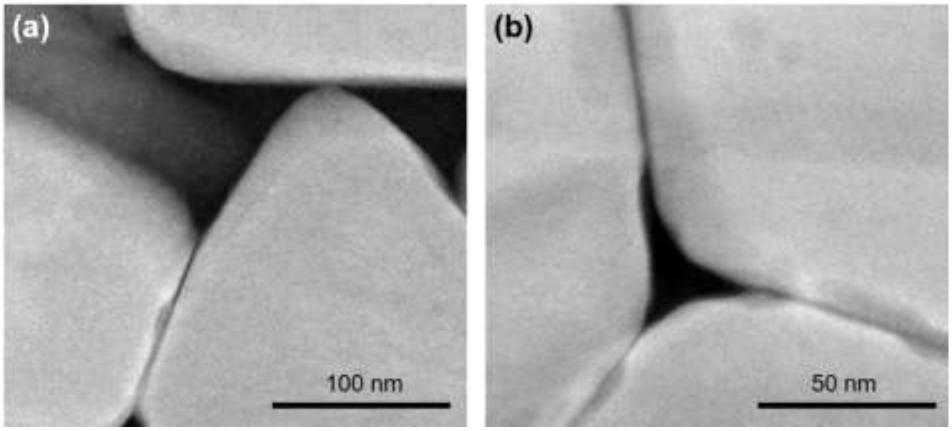

Figure S6 HAADF-STEM images of pristine NMC811 cathode.

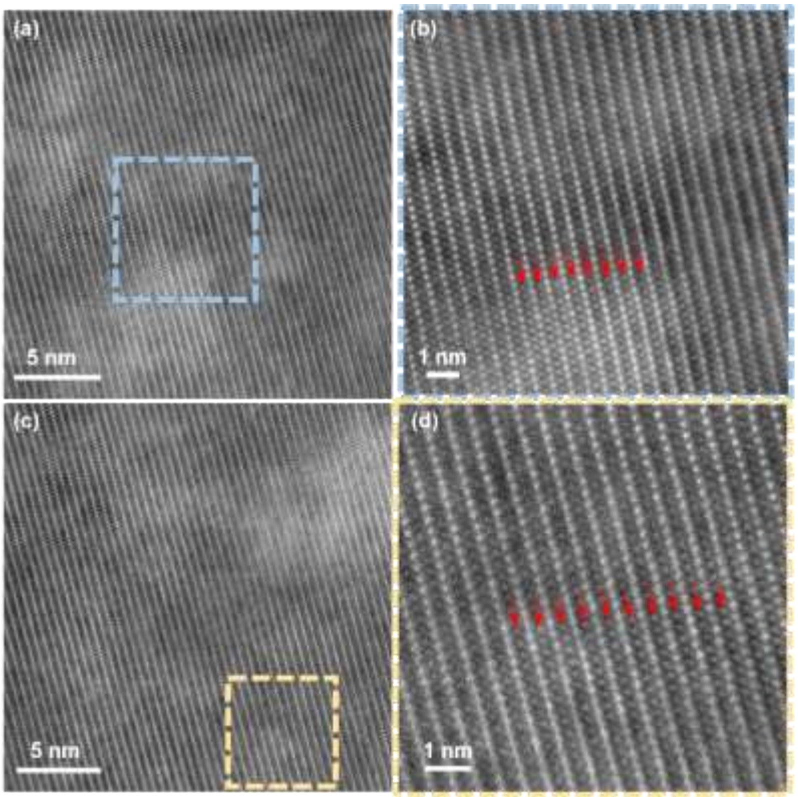

Figure S7 STEM images showing the bulk structure of 2C-charged NMC811 with arrows indicating the disordered domains.

Table S1. Crystallographic parameters of pristine NMC811 refined by Rietveld method

Atom	Site	x	y	z	Fraction
Li1	3a	0	0	0	0.9564
Ni1	3a	0	0	0	0.0419
Li2	3b	0	0	0.50	0.0436
Ni2	3b	0	0	0.50	0.7581
Co	3b	0	0	0.50	0.1000
Mn	3b	0	0	0.50	0.1000
O	6c	0	0	0.24	0.9832
a=b=2.87327 Å c=14.21264 Å V=101.615 Å^3					

R_p=4.03%, GOF=1.54

Table S2. Crystallographic parameters of 0.2C-charged NMC811 refined by Rietveld method

Atom	Site	x	y	z	Fraction
Li1	3a	0	0	0	0.9437
Ni1	3a	0	0	0	0.0538
Li2	3b	0	0	0.50	0.0563
Ni2	3b	0	0	0.50	0.7462
Co	3b	0	0	0.50	0.1000
Mn	3b	0	0	0.50	0.1000
O	6c	0	0	0.24	0.9276
a=b=2.86276 Å c=14.24722 Å V=101.118 Å^3					

R_p=4.86%, GOF=1.97

Table S3. Crystallographic parameters of 0.5C-charged NMC811 refined by Rietveld method

Atom	Site	x	y	z	Fraction
Li1	3a	0	0	0	0.9389
Ni1	3a	0	0	0	0.0960
Li2	3b	0	0	0.50	0.0611
Ni2	3b	0	0	0.50	0.7040
Co	3b	0	0	0.50	0.1000
Mn	3b	0	0	0.50	0.1000
O	6c	0	0	0.24	0.9296
a=b=2.84901 Å c=14.2907 Å V=100.455 Å^3					

R_p=7.82%, GOF=2.85

Table S4. Crystallographic parameters of 1C-charged NMC811 refined by Rietveld method

Phase	NMC811						Al					
Fraction	0.9327						0.0673					
	Atom	Site	x	y	z	Fraction	Atom	Site	x	y	z	Fraction
	Li1	3a	0	0	0	0.9218	Al		0	0	0	1
	Ni1	3a	0	0	0	0.0989						
	Li2	3b	0	0	0.50	0.0782						
	Ni2	3b	0	0	0.50	0.7011						
	Co	3b	0	0	0.50	0.1000						
	Mn	3b	0	0	0.50	0.1000						
	O	6c	0	0	0.24	0.9612						
	a=b=2.84079 Å c=14.3123 Å V=100.027 Å^3						a=b=c=4.0494 Å V=66.401 Å^3					
	R_p=10.38%, GOF=3.85											

Table S5. Crystallographic parameters of 2C-charged NMC811 refined by Rietveld method

Atom	Site	x	y	z	Fraction
Li1	3a	0	0	0	0.9438
Ni1	3a	0	0	0	0.0095
Li2	3b	0	0	0.50	0.0562
Ni2	3b	0	0	0.50	0.7905
Co	3b	0	0	0.50	0.1000
Mn	3b	0	0	0.50	0.1000
O	6c	0	0	0.24	0.9383
a=b=2.82898 Å c=14.37623 Å V=99.640 Å^3					
R_p=4.86%, GOF=1.93					
